# PPAR Gamma and Angiogenesis: Endothelial Cells Perspective

**DOI:** 10.1155/2016/8492353

**Published:** 2016-12-07

**Authors:** Jerzy Kotlinowski, Alicja Jozkowicz

**Affiliations:** ^1^Department of General Biochemistry, Faculty of Biochemistry, Biophysics and Biotechnology, Jagiellonian University, Krakow, Poland; ^2^Department of Medical Biotechnology, Faculty of Biochemistry, Biophysics and Biotechnology, Jagiellonian University, Krakow, Poland

## Abstract

We summarize the current knowledge concerning PPAR*γ* function in angiogenesis. We discuss the mechanisms of action for PPAR*γ* and its role in vasculature development and homeostasis, focusing on endothelial cells, endothelial progenitor cells, and bone marrow-derived proangiogenic cells.

## 1. Angiogenesis

**Figure 1:**
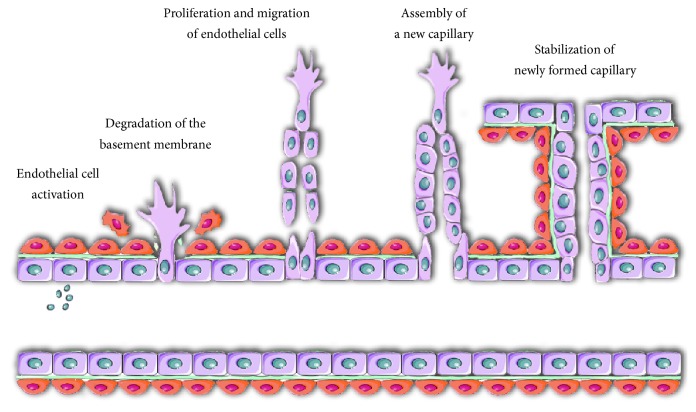
Angiogenesis. Development of a new capillary from preexisting blood vessel is called angiogenesis. Angiogenesis is a multistage process that requires activation of endothelial cells by angiogenic growth factors (e.g., vascular endothelial growth factor and basic fibroblast growth factor), followed by degradation of the basement membrane. Next, endothelial cells proliferate and migrate to assemble into tubes. Finally, they deposit a new basement membrane, secrete cytokines (e.g., platelet derived growth factor and angiopoietins) to attract supporting cells, which in turn stabilize new vessels.

In human embryos, the development of vasculature starts at day 21 after conception. The de novo formation of blood vessels occurs from cells called angioblasts, which form tubes in a process known as vasculogenesis. Angioblasts proliferate and generate the very first vascular plexus, which in turn grows and expands* via* angiogenesis [1]. Angiogenesis refers to the sprouting of endothelial cells from preexisting vessels as well as their migration and proliferation to create new tube-like structures [2]. During angiogenesis, one can distinguish between several well-characterized stages. After the activation of endothelial cells by angiogenic growth factors (e.g., vascular endothelial growth factor (VEGF) and basic fibroblast growth factor (bFGF)), ECs degrade the basement membrane and then proliferate and migrate to assemble into tubes. Finally, they deposit a new basement membrane, secrete cytokines (e.g., platelet derived growth factor (PDGF) and angiopoietins) to attract supporting cells, which in turn stabilize new vessels [3]. Such newly generated vessels can further grow or undergo remodeling* via* intussusception, which describes the generation of new capillaries by the splitting of preexisting ones (Figure 1). Angiogenesis is not restricted to embryonic development but also takes place in adults, where it is necessary for both physiological and pathological processes. Normal physiological processes that involve angiogenesis include the female reproductive cycle, wound healing, bone repair, postischemic repair, and hair growth [1]. Importantly, excessive angiogenesis is a hallmark of diseases such as cancer, proliferative retinopathy, psoriasis, or rheumatoid arthritis [4, 5]. By contrast, insufficient blood vessel formation can lead to the development of nonhealing ulcers and myocardial or brain ischemia [5, 6].

**Figure 2:**
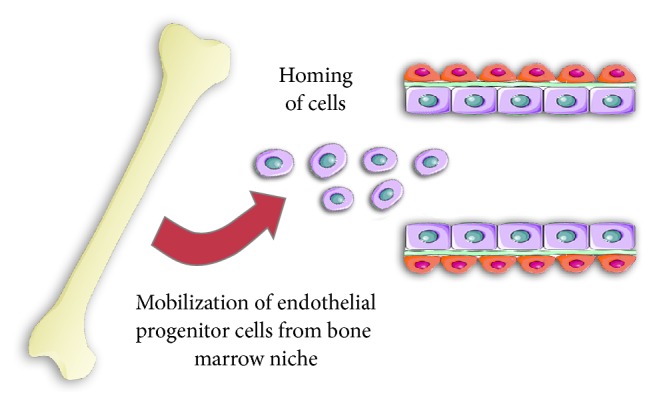
Endothelial progenitor cells and angiogenesis. EPCs were described as cells that are present in the bone marrow niche, from where, in response to injury or hypoxia, they are released into the blood and mobilized to the injured tissue. View of EPCs mode of action changes over time and today scientists postulate that their complex nature needs more studies; please see main text for more details.

For many years it was thought that the de novo formation of blood vessels from undifferentiated precursor cells occurs only during fetal development. It was believed that, in adults, the regeneration and formation of new blood vessels relied on the migration and differentiation of mature ECs. This widely accepted view has changed since the discovery by Asahara and coworkers of endothelial progenitor cells (EPCs) in the blood of adults that are capable of proliferation, migration, and incorporation into existing vessels. Isolated from the blood of adult volunteers, CD34+/VEGFR-2+ cells were grown in vitro, and after several days they began to express other endothelial-specific markers such as CD31, E-selectin, Tie-2, and eNOS [7]. Next, experiments carried out by the same group confirmed the participation of EPCs released from bone marrow in the formation of blood vessels, both under physiological (endometrial hyperplasia, blood supply to the corpus luteum, and wound healing) and pathological conditions (tumor growth, myocardial infarction, or ischemic hind limb) [8, 9]. However, despite very promising initial reports describing angiogenic properties for EPCs, researchers have failed to identify a specific antigen profile that uniquely characterizes such EPCs. As a result, a variety of protocols for EPC isolation, growth, and characterization are used [10]. Today, despite 19 years of studying EPCs, there is a growing number of contradictory conclusions with respect to their role in the cardiovascular system. These discrepancies are mainly due to the strong phenotypic overlap between EPCs and circulating proangiogenic cells from the hematopoietic lineage, a lack of universal data reporting, and—as reported before—differing definitions of the studied cell populations [11]. It was thought that EPCs are present in the bone marrow niche, from where, in response to injury or hypoxia, they are released into the blood and mobilized to the injured tissue [9, 12, 13]. It was originally postulated that, after entering a damaged or ischemic tissue, EPCs stimulate the formation of blood vessels by differentiating into mature endothelial cells (Figure 2). They could also directly incorporate into existing damaged vessel structures, filling gaps in the endothelial layer [7]. However, due to conflicting results concerning the participation of EPCs in already formed vessels (from 0% to 80% of endothelial cells), it is difficult to determine the true importance of this process [14–20]. Moreover, it is clear that the cell surface antigens and colony assays used to identify EPCs have significant overlap with those of cells from the hematopoietic lineage. Such hematopoietic subsets (e.g., CD31^+^CD34^bright^CD45^dim^AC133^+^) also circulate in the blood and participate in vascular repair and regeneration [21].

It seems, however, that paracrine stimulation of blood vessel regeneration might be the key mechanism of EPCs (or bone marrow-derived proangiogenic cells) action. Proangiogenic cytokines that are produced at high levels by EPCs include interleukin-8 (IL-8), hepatocyte growth factor (HGF), VEGF, platelet derived growth factor-BB (PDGF), bFGF, and monocyte chemoattractant protein-1 (MCP-1) [22]. Importantly, intramuscular administration of conditioned media collected from EPCs improved blood flow regeneration in an ischemic rat hind limb model. Our recent data also proved that conditioned media is more efficient than the injection of cells for restoring blood perfusion in a mouse ischemic limb model. It was also noteworthy that, in this model, approximately 70% of the injected cells were eluted from the site of injection within first 6 hours, and most remaining cells died during first three days after injection [23, 24].

## 2. Role of PPAR**γ** in Vascular Development

PPAR*γ* was identified for the first time in murine adipose tissue, although in humans its cDNA was first isolated from hematopoietic cells [25, 26]. Today we know that it belongs to the nuclear receptor superfamily, which are ligand-activated transcription factors that drive specific gene expression programs upon stimulation with a ligand (for review about PPAR*γ* mechanism of action please see [27, 28]). PPAR*γ* is most abundantly expressed in adipose tissue, where it regulates adipocyte maturation [29, 30]. It is also involved in the regulation of lipid metabolism, as ligand-dependent activation leads to increased fatty acid uptake and storage. Furthermore, PPAR*γ* plays an important role in glucose homeostasis as an insulin-sensitizing agent, which is why agonists of PPAR*γ* are currently used to treat diabetes. Although PPAR*γ* was initially found to be critical for adipocyte differentiation and function, over time, it was also found to play an important role in the cardiovascular system. Importantly, its activity has been demonstrated in the vessel wall, both in endothelial cells (ECs) and in vascular smooth muscle cells (VSMCs), suggesting its vital role in angiogenesis [31, 32].

**Figure 3:**
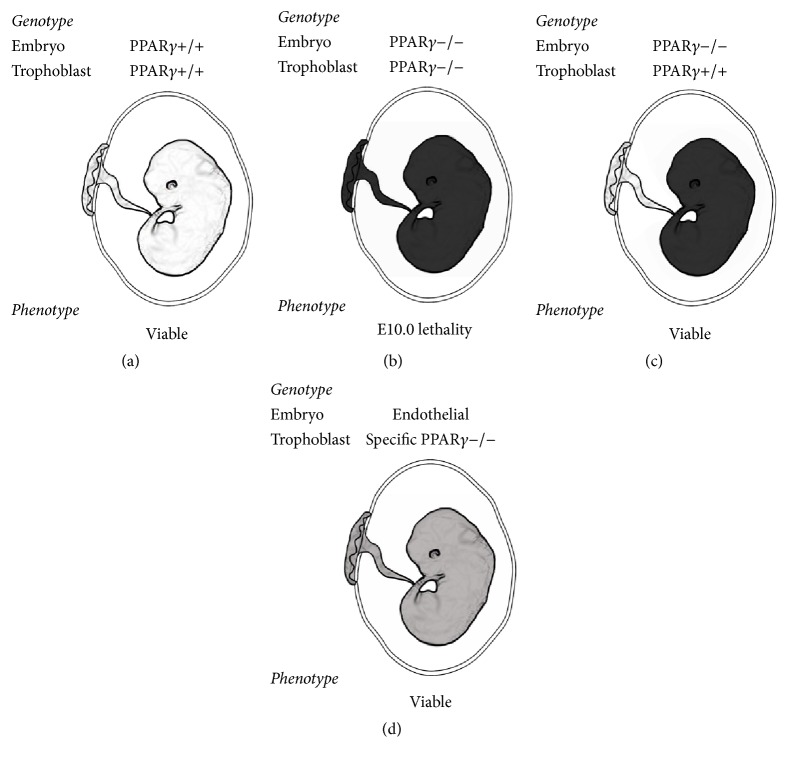
Role of PPAR*γ* in vascular development. (a) Expression of PPAR*γ* (PPAR*γ*+/+) is necessary for proper angiogenesis during embryogenesis. (b) PPAR*γ* knockout (PPAR*γ*−/−) mice are embryonically lethal by E10.0 due to the impaired placental vascularization. (c) Deletion of PPAR*γ* in the embryo, but not in the trophoblast, or (d) specifically in the endothelial cells does not disturb angiogenesis and leads to the generation of viable pups.

The importance of PPAR*γ* in angiogenesis was demonstrated by the generation of knockout animals in 1999. As reported by Barak and coworkers, PPAR*γ* null mice are embryonically lethal by E10.0, due to placental dysfunction characterized by defective trophoblast differentiation and markedly impaired placental vascularization (Figures 3(a) and 3(b)) [33]. Moreover, supplementation of PPAR*γ*−/− embryos with wild-type placentas resulted in an apparently normal vascular system during further embryogenesis, although the pups died some days after birth due to combination of pathologies, including severe lipodystrophic changes and hemorrhages [33]. In a latter study, to rescue the embryonic lethality of global PPAR*γ* knockout embryos, floxed PPAR*γ* mice were crossed with Mox2-Cre mice to inactivate PPAR*γ* in the embryo but not in trophoblasts (Figure 3(c)). Such an approach allowed for the generation of viable PPAR*γ*−/− animals that were characterized by lipodystrophy, insulin resistance, and hypotension [34]. These mice showed increased endothelium-dependent relaxation in response to acetylcholine, which was not associated with changes in eNOS expression or phosphorylation [34]. Mice in which PPAR*γ* function was selectively knocked out only in endothelial cells—based on the same Cre-Lox system and Tie2-Cre construct—were phenotypically indistinguishable from wild-type littermates (Figure 3(d)) [35]. However, when Tie2-Cre transgenic mice were fed high-fat diets, they had significantly elevated systolic blood pressure that was not observed after a normal diet or salt-loading [35]. This observation could be at least partially explained by data showing that intact aortic segments from endothelial-specific PPAR*γ*−/− mice released less nitric oxide than those from controls [36]. Importantly, disruption of endothelial PPAR*γ* contributes to endothelial dysfunction in vivo, as reduced nitric oxide production in PPAR*γ*−/− aortas was associated with increased oxidative stress and enhanced activation of NF*κ*B in aortic homogenates [36]. The results mentioned above indicate that tight regulation of PPAR*γ* expression is crucial for proper angiogenesis. Importantly, PPAR*γ*−/− mice displayed defects in vasculature structure as well as a lack of balance between pro- and antiangiogenic factors [33, 37]. Consistent with this data, McCarthy and coworkers demonstrated that administration of a PPAR*γ* antagonist (T0070907) to pregnant rats led to endothelial dysfunction, reduced expression of VEGF, and increased levels of plasma soluble VEGF receptor-1 (sVEGFR-1), which acts as a VEGF scavenger [38]. Interestingly, the treatment of pregnant wild-type mice with rosiglitazone also resulted in a disorganization of placental microvasculature [37]. However, our recently published data indicate that angiogenesis in wound healing and hind limb ischemia models is not affected by an ~50% decrease in the expression of PPAR*γ* in normoglycemic PPAR*γ*+/− mice [24].

## 3. PPAR**γ** and Endothelial Cells

**Figure 4:**
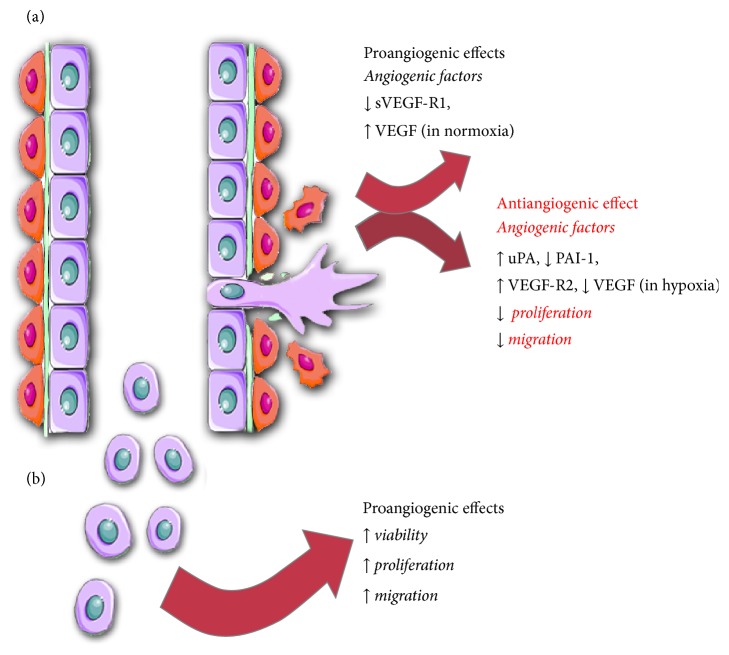
Effects of PPAR*γ* activation on angiogenesis. (a) Angiogenic properties of endothelium is decreased upon activation of PPAR*γ via* reduction of migratory properties and inhibited proliferation and by diminished production of angiogenic factors. (b) Stimulation of PPAR*γ* in diabetic endothelial progenitor cells induces angiogenesis. Detailed description of molecular mechanisms responsible for observed effects is in the main text.

The first evidence of PPAR*γ* expression in ECs came from studies examining the influence of PPAR*γ* activation on angiogenic and apoptotic properties of ECs [31, 39]. Today, after many years of study, we know that PPAR*γ* is a very important regulator of EC biology that is involved in the regulation of angiogenesis at a variety of stages. PPAR*γ* activation was shown to influence the production of cytokines by ECs as well as their proliferation, migration, and ability to form capillaries, although certain studies showed variable results (Figure 4(a)).

## 4. Angiogenic Factors

Degradation of the extracellular matrix is a necessary step during early stages of angiogenesis. Among the various proteases produced by ECs that are involved in capillary growth are urokinase plasminogen activator (uPA) and the matrix metalloproteinases (MMPs). The primary physiological substrate of uPA is plasminogen, which is an inactive form of serine protease plasmin. The first evidence for modulation of the expression of uPA and its inhibitor (PAI-1, plasminogen activator inhibitor-1) by PPAR*γ* came from studies performed in 1999 by Xin and coworkers. They observed that treatment of HUVECs with 15d-PGJ2 reduced the mRNA levels of uPA and increased the levels of PAI-1 mRNA [31]. Subsequent experiments showed that treatment of HUVECs with PPAR*α* and PPAR*γ* activators (linolenic acid, linoleic acid, oleic acid, and PGJ2) augmented PAI-1 mRNA expression and protein secretion in a concentration-dependent manner [40]. Similarly, our data proved that activation of PPAR*γ* by 15d-PGJ2 could potently inhibit the synthesis of uPA in HMEC-1 cells. Importantly, this effect was also reproduced by the treatment of cells with troglitazone, suggesting PPAR*γ*-dependent action [41].

By contrast, the opposite effect was also reported in control and TNF-stimulated ECs. TZDs decreased basal and TNF-stimulated PAI-1 secretion and mRNA expression in HUVECs in a dose-dependent fashion [42, 43]. As shown by Liu and coworkers, TZDs inhibited the induction of PAI-1 by TNF, although the specific PPAR*γ* inhibitor SR-202 failed to modulate this effect. Moreover, ECs transfected with a dominant-negative PPAR*γ* construct exhibited the same phenotype [43].

The most commonly used classification of MMPs is based on their substrate specificity and cellular localization, dividing them into collagenases, gelatinases, stromelysins, and membrane-type MMPs. We found that treatment of HMEC-1 with 15d-PGJ2 increased the synthesis of MMP-1 protein but that TZDs (ciglitazone and troglitazone) did not influence MMP-1 production, arguing against the involvement of PPAR*γ*. Importantly, stimulatory effects were reversed by NAC treatment, suggesting that 15d-PGJ2 upregulates MMP-1 expression in HMEC-1 cells through the induction of oxidative stress [44]. As shown by Park and coworkers, the antiangiogenic activity of troglitazone in ECs is correlated with the suppression of VEGF-induced MMP-2 and membrane-type 1-MMP expression and is also linked to ROS generation. Effects of troglitazone on VEGF-induced MMP-2 and MT1-MMP expression were abolished after addition of the NADPH oxidase inhibitor diphenylene iodium or the ERK inhibitor PD98056 [45]. In human brain microvascular endothelial cells, Huang and coworkers showed that the addition of exogenous PPAR*γ* agonists resulted in downregulation of MMP-2 and MMP-9 expression as well as their proteasome activities [46]. In addition, activation of PPAR*γ* with ciglitazone in mouse aortic vascular endothelial cells reduced MMP-9 activation [47].

Vascular endothelial growth factor (VEGF) is a crucial inducer of blood vessel formation during embryogenesis and in postnatal life. VEGF acts as a specific survival factor for ECs, regulating many endothelial functions, such as proliferation, migration, morphogenesis, vascular permeability, and the production of vasoactive molecules [48]. The first report concerning the potential influence of PPAR*γ* ligands on VEGF action came from a study performed in 1999, where it was reported that treatment of HUVECs with 15d-PGJ2 reduced the mRNA levels of vascular endothelial cell growth factor receptors 1 and 2 [31]. We and others confirmed this finding, showing that PPAR*γ* activation results in reduced expression of VEGF-R2 and soluble VEGF-R1 [49, 50]. Consistent with these results, inhibition of PPAR*γ* in pulmonary arterial endothelial cells resulted in upregulation of VEGF-R2 [51]. By contrast, in one study, VEGF-R2 expression was found to be enhanced in response to PPAR*γ* activation by troglitazone and attenuated by GW9662, a specific antagonist of PPAR gamma [52].

Transcriptional upregulation of VEGF was reported for the first time in rat and human vascular smooth muscle cells stimulated in vitro with ciglitazone or rosiglitazone [53, 54]. In ECs, activation of PPAR*γ* was also shown to influence VEGF production. We have shown that treatment of HMEC-1 cells with 15d-PGJ2 significantly and dose-dependently increased VEGF promoter activity, mRNA expression, and protein secretion. By contrast, addition of ciglitazone caused a much weaker induction, suggesting a primarily PPAR*γ*-independent action. Cells treated with 15d-PGJ2 were characterized by augmented expression of heme oxygenase-1 (HO-1) protein. As inhibition of the HO-1 pathway significantly reduced the stimulatory effects of 15d-PGJ2 on VEGF synthesis, we postulated that the upregulation of VEGF expression in response to 15d-PGJ2 in HMEC-1 is mediated by activation of HO-1 [55]. Later experiments showed that the proangiogenic activity of HO-1 in ECs can be mimicked by the addition of carbon monoxide releasing molecules [41]. Also of note, we demonstrated that the regulation of VEGF by PPAR*γ* ligands is dependent on oxygen concentration. Under hypoxia, in contrast to normoxia, induction of PPAR*γ* by 15d-PGJ2 decreases VEGF synthesis through inhibition of the HIF-1 pathway [56]. More recently, Biscetti and coworkers showed that activation of PPAR*α* and PPAR*γ* in ECs leads to enhanced tube formation, which was associated with increased production of VEGF [57].

Further confirmation of PPAR*γ*-independent activation of VEGF by PPAR*γ* ligands came from in vivo studies. Using a hind limb ischemia murine model, Biscetti and coworkers found that pioglitazone enhanced the restoration of blood flow and capillary density in ischemic muscles and that this process is associated with increased expression of VEGF. However, direct activation of PPAR*γ* by GW1929 did not restore blood flow recovery, in contrast to combined treatment with pioglitazone and GW9662 (the selective PPAR*γ* inhibitor) suggesting a PPAR*γ*-independent action. Importantly, these beneficial effects were abrogated upon endogenous Akt inhibition [58].

## 5. Proliferation

Fukunaga and coworkers evaluated basal proliferation in endothelial cells isolated from different human vascular beds (aorta, carotid artery, and umbilical vein) as well as from the bovine carotid artery. When these cultured endothelial cells were treated daily with troglitazone or pioglitazone for 5 days at a 10 nmol/L dose, both compounds induced the proliferation of ECs. By contrast, activation of PPAR*γ* with higher concentrations (10 *μ*mol/L) significantly suppressed DNA synthesis [59]. The inhibition of EC proliferation was also observed in HUVECs overexpressing PPAR*γ* or wild-type cells stimulated with troglitazone alone. A combination of PPAR*γ* overexpression and troglitazone treatment resulted in a further decrease in the thymidine uptake by HUVECs [60]. VEGF-induced EC proliferation was also inhibited by other PPAR*γ* agonists, including pioglitazone, ciglitazone, troglitazone, and 15d-PGJ2. Although all tested compounds exerted the same effect, 15d-PGJ2 reduced EC proliferation much more potently than the TZDs [49, 61]. Activation of PPAR*γ* by 15d-PGJ2 was also shown to reduce only partially and transiently the expression of VEGFR-1 and VEGF-R2 in HUVEC, which was insufficient to fully explain the observed results. Additionally, stimulation with 15d-PGJ2 decreased the activities of c-Jun and c-Myc, and overexpression of c-Myc attenuated its antiproliferative effects in HUVEC [49]. In a recently published paper, a novel PPAR*γ* agonist KR-62980 also inhibited VEGF-induced HUVEC proliferation. KR-62980 downregulated VEGF-induced VEGFR-2 expression but increased the expression of phosphatase and tensin homolog deleted on chromosome 10 (PTEN) in parallel with reduced phosphorylation of extracellular signal-related kinase 1/2 (ERK1/2) and p38 MAPK [50]. Another explanation of the antiproliferative effects of PPAR*γ* activation came from studies performed by Sheu and coworkers, who demonstrated endothelial arrest at G1 phase after treatment with rosiglitazone. Rosiglitazone inhibited endothelial proliferation in a dose-dependent manner by decreasing the production and activity of several key cell cycle regulators (cyclin D1, A, E, cdk2, and cdk4, hypophosphorylation of Rb) that control G1/S progression [62]. Finally, another group confirmed that troglitazone significantly inhibits serum-induced proliferation of HUVECs in a concentration-dependent manner through the suppression of casein kinase 2 [63].

## 6. Migration

PPAR*γ* ligands are also involved in inhibiting EC migration. VEGF-induced migration of HUVECs is inhibited by troglitazone and ciglitazone through the inhibition of Akt phosphorylation [64]. By contrast, induction of EC migration by leptin was shown to depend on the PI3K/Akt and ERK1/2 MAPK pathways [65]. The authors demonstrated that the antimigratory effects of PPAR*γ* activation by troglitazone or ciglitazone were due to the inhibition of Akt but not the ERK 1/2 kinases. The inhibition of Akt phosphorylation by TZDs was accompanied by upregulation of PTEN, a phosphatase that functions as a negative regulator of PI3K/Akt signaling [65, 66]. In another study, using scrape-wound and chemotactic assays, researchers found that troglitazone dose-dependently inhibited the migration and proliferation of cultured macrovascular endothelial cells in low-glucose (5 mmol/L) and high-glucose (25 mmol/L) media [67]. VEGF-induced EC migration is also inhibited by ciglitazone, 15d-PGJ2, and a novel PPAR*γ* ligand, KR-62980 [49, 50, 68]. As shown by Aljada and coworkers, EC migration through an 8-*μ*m pore filter to a feeder layer containing vitronectin as a chemoattractant was significantly blunted by pioglitazone or rosiglitazone treatment [68]. As described earlier, rosiglitazone markedly decreased VEGF-induced tube formation and endothelial cell migration in a wound-healing migration assay, which could have been due to disorganization of the actin cytoskeleton [62]. Similarly, troglitazone significantly suppressed VEGF-induced cell proliferation and invasion of HUVECs into the Matrigel basement membrane, which was not reversed by GW9662 [45].

## 7. Angiogenesis Tests

The most popular in vitro angiogenesis tests rely on the ability of ECs to form tube-like structures when plated on top of a reconstituted basement membrane extracellular matrix (e.g., Matrigel). Such a differentiation process involves steps that are similar to what occurs in vivo during blood vessel formation, including cell adhesion, migration, alignment, protease secretion, and tubule formation [69]. As shown for the first time in 1999, activation of PPAR*γ* by specific ligands, such as 15d-PGJ2, BRL49653, or ciglitazone, dose-dependently suppressed HUVEC differentiation into tube-like structures [70]. Consistent with this data, Murata and coworkers reported that incubation of bovine choroidal endothelial cells with rosiglitazone or troglitazone significantly inhibited not only VEGF-induced tube formation but also migration and proliferation [71]. In addition, our experiments proved that PPAR*γ* activation (by rosiglitazone or 15d-PGJ2) diminished the angiogenic potential of ECs plated not only on Matrigel but also in a three dimensional spheroid test [49]. We used ECs embedded in collagen gel because they can generate radial capillaries that more closely resemble angiogenesis in vivo. More recently, Aljada and coworkers used a chick chorioallantoic membrane (CAM) model to evaluate the efficacy of pioglitazone and rosiglitazone on VEGF and bFGF-induced angiogenesis. The TZDs used in that study significantly inhibited the proangiogenic effects of bFGF and VEGF in the CAM model [68]. As demonstrated by Park and coworkers, the antiangiogenic actions of TZDs in the CAM model were PPAR*γ* independent, as the observed phenotype was not reversed by treatment with PPAR*γ* antagonists, GW9662, or bisphenol A diglycidyl ether. Additionally, troglitazone blocked VEGF-induced ROS production and ERK phosphorylation, and again this inhibitory effect was not reversed by GW9662. NADPH oxidase or ERK inhibition mimicked effects obtained with troglitazone, suggesting that inhibition of angiogenesis by troglitazone is mediated by ROS production and ERK phosphorylation [45].

Although the antiangiogenic activity of PPAR*γ* agonists has been described in many papers, there is also some evidence for opposing effects. As mentioned above, 15d-PGJ2 induced expression of VEGF and IL-8 in ECs, but this action was PPAR*γ* independent [55, 56, 72]. Recently, Fujii and coworkers demonstrated that, in HUVECs, VEGFR-2 expression was enhanced in response to PPAR*γ* activation by troglitazone and attenuated by GW9662, a specific inhibitor. In the same cells, endothelial morphogenesis measured in a tube formation assay was also stimulated by troglitazone and inhibited by GW9662, indicating that PPAR*γ* activation positively mediates angiogenesis [52]. EC proliferation was also induced with low concentrations of TZDs [59]. The reasons for these discrepancies are not clear. It is possible that the concentrations of PPAR*γ* ligands used in some experiments were extremely high and therefore proapoptotic [45, 50]. It is also possible that PPAR*γ* ligands exert different effects on ECs from different vascular beds due to the heterogenous expression of PPAR*γ*-regulated genes. CD36 is one such example of a PPAR*γ*-dependent gene that is upregulated by PPAR*γ* agonists. CD36 encodes a scavenger receptor that among many ligands binds antiangiogenic thrombospondin-1 [73]. CD36 receptor is mainly expressed in microvascular endothelial cells and at lower level in the venous endothelium [74]. What is more, even within the microvasculature, the expression of CD36 is organ specific, with the highest levels in the heart, muscles, and lungs and the lowest levels in the bone marrow [75]. Therefore, it is possible that such differences in expression could modulate the angiogenic response to PPAR*γ* agonists.

## 8. PPAR**γ** and Endothelial Progenitor Cells

As mentioned before, vascular repair and angiogenesis depend both on mature endothelial cells and endothelial progenitors [76, 77]. However, the phenotypic characterization of EPCs is still the subject of lively debate and controversy. There is also no commonly accepted standardized method for EPC isolation and culturing, which complicates the interpretation of results. Indeed, rigorous comparisons of recently described methods confirmed that the term “EPCs” as it is used today does not precisely define one cell population [10, 20]. In addition, characterizing EPCs is made difficult by the lack of specific antigens, as the markers used for their immunophenotyping are also expressed on surface of ECs and HSCs. Finally, the use of different isolation protocols, different culture conditions, and even different markers for EPC characterization makes it difficult to compare results obtained by different research groups. For these reasons, in our studies, we use term EPCs only for cells analyzed by flow cytometry with strictly a defined phenotype (CD45−/Sca-1+/KDR+), whereas we refer to cells isolated and expanded in vitro as a bone marrow-derived proangiogenic cells (PACs) [24, 78].

Despite the lack of standardized definitions, analyses have consistently indicated that the angiogenic properties of EPCs/PACs are impaired in type 2 diabetes, including decreased cell numbers, decreased mobilization, and decreased angiogenic potential [12, 79–82]. Importantly, such properties could be improved using PPAR*γ* activation (Figure 4(b)) [13, 83–86]. Experiments evaluating the impact of TZDs on EPC function were first described by Pistrosch et al. in 2005. The authors described results of a three-month therapy program involving rosiglitazone in patients with diabetes resulting in the normalization of impaired EPC migration and numbers, and beneficial effects persisted up to 9 weeks after treatment [83]. Similar results were obtained in patients enrolled in a combined antidiabetic therapy with pioglitazone and metformin. Both the direct and indirect effects of pioglitazone on EPCs increased their number and proliferation status and improved their migration and adhesion to fibronectin or collagen [84]. Activation of PPAR*γ* with rosiglitazone also attenuated the negative effects of advanced glycation end products (AGEs), and the stimulation of EPCs decreased inhibition of proliferation, migration, and activation of Akt and eNOS phosphorylation induced by AGEs [85, 87].

Intensive studies analyzing impact of TZDs on EPC biology has revealed some of the molecular mechanisms responsible for this phenomenon. As shown by Gensch and coworkers, administration of pioglitazone to mice not only increased the number of EPCs but also activated telomerase in endothelial cells via induced expression of telomere binding agent, type 2 (telomere repeat-binding factor 2, tert-2) [88]. Similar results were reported for human cells, as the activation of telomerase by pioglitazone in cultivated EPCs was prevented by Akt inhibitors [89]. It was also found that treatment with rosiglitazone facilitated reendothelialization in diabetic patients through decreased ROS generation and improved bioavailability of nitric oxide (NO) in endothelial cells [90].

Our recently published results showed that PACs derived from db/db mice displayed impaired migration and showed the formation of cord-like structures on Matrigel and capillary outgrowth from spheroids. Only the proliferation rate was not significantly affected [24]. Our data are in accordance with other studies showing decreased angiogenic potential in diabetic EPCs isolated from both humans and rodents [12, 79–82]. A paper published by Liang et al. demonstrated that AGEs, which are present in diabetes, induce EPC apoptosis and impair SDF-1 and NO production [85]. Transcriptome analysis suggested that the impaired migration of PACs can be associated with upregulation of integrins and the concomitant downregulation of genes involved in filopodia formation, such as efexin or CCT2 [24, 91]. Furthermore, PACs isolated from db/db mice showed decreased paracrine potential, which could be related to decreased expression of VEGF-C, VEGF-D, FGF7, or angiogenin. Incubation of PACs with rosiglitazone upregulated VEGF and KC protein levels in wild-type and db/db PACs but had no effect on VEGF-R1 or VEGF-R2 expression [24].

Importantly, we proved a direct effect for PPAR*γ* activation on the angiogenic potential of PACs, as the incubation of cells with rosiglitazone in vitro enhanced migration, the formation of cord-like structures and capillary outgrowth. These effects were PPAR*γ*-dependent, as demonstrated* via* preincubation of cells with GW9662, an PPAR*γ* antagonist [24]. A similar increase in the migratory capacities of cultured bone marrow-derived cells was observed earlier after treatment with pioglitazone or troglitazone [87, 88], which could be mediated by reduced expression of ICAM-1 and VCAM-1 adhesion molecules upon PPAR*γ* activation [86].

Beneficial effects of TZDs on EPCs have also been shown in patients with normal glucose tolerance but who are suffering from ischemic heart disease. Treatment for 30 days with pioglitazone increased not only the number of CD34+/KDR+ cells but also their clonogenic potential and SDF-1-induced migration in a PPAR*γ*-dependent manner [87]. Importantly, PPAR*γ* agonists improved endothelial functions and angiogenic capacities independently of insulin sensitization in normoglycemic patients [92]. Normoglycemic humans were treated for 6 weeks with rosiglitazone (8 mg once daily) or placebo and then adipose tissue vascularization was evaluated, with the authors reporting increased capillary density and angiogenic potential in patients receiving TZD [92].

PPAR*γ* activation was also reported to be beneficial in EPCs isolated from the bone marrow of normoglycemic rats. Pioglitazone prevented apoptosis* via* the PI3K/Akt signaling pathway [13]. Another study proved that a dual PPAR*α*/*γ* agonist, aleglitazar, administered to normoglycemic mice at a 10 mg/kg/day dose increased the number of Sca-1/VEGFR2 double-positive cells in bone marrow and peripheral blood. Aleglitazar also improved cell migration and enhanced neoangiogenesis. Importantly, cells isolated from healthy donors treated in vitro with aleglitazar were characterized by reduced oxidative stress-induced apoptosis and p53 expression, whereas the phosphorylation of eNOS and Akt was elevated [93].

## 9. Conclusions and Future Perspectives

PPAR*γ* originally described as a transcription factor regulating expression of genes involved in carbohydrate and lipid metabolism has been more recently studied in the context of cardiovascular system. Of great importance, its activity has been reported in vessel wall, in both endothelial cells and vascular smooth muscle cells. Because endothelium is a master regulator of angiogenesis, we attempted to summarize PPAR*γ* roles in this process focusing on endothelial cells.

Activation of PPAR*γ* shows predominantly antiangiogenic properties. There is also some evidence for the opposite effects, but 15d-PGJ2 mediated induction of VEGF and IL-8 expression in ECs turned out to be PPAR*γ* independent. Besides decreasing expression of angiogenic factors, PPAR*γ* activation leads to the reduced migration and proliferation of ECs. Additionally, an inhibitory net effect of PPAR*γ* induction was demonstrated in various angiogenic tests employing endothelial cells. During the past decade, accumulating evidence about circulating proangiogenic progenitors has considerably improved our understanding of PPAR*γ* actions. In contrast to mature ECs, activation of PPAR*γ* improves angiogenic potential of EPCs. These effects are described predominantly for cells isolated from diabetic patients; therefore it might be difficult to distinguish the direct effect of PPAR*γ* activation from the indirect results of improved glycaemic control. Finally, while studying PPAR*γ* activation by its ligands, one should not forget to differentiate between PPAR*γ* dependent and PPAR*γ*-independent actions, for example by using both PPAR*γ* agonists and antagonists.

## References

[1] Carmeliet P. (2005). Angiogenesis in life, disease and medicine. *Nature*.

[2] Madeddu P. (2005). Therapeutic angiogenesis and vasculogenesis for tissue regeneration. *Experimental Physiology*.

[3] Coulon C., Georgiadou M., Roncal C., De Bock K., Langenberg T., Carmeliet P. (2010). From vessel sprouting to normalization: role of the prolyl hydroxylase domain protein/hypoxia-inducible factor oxygen-sensing machinery. *Arteriosclerosis, Thrombosis, and Vascular Biology*.

[4] Bellou S., Pentheroudakis G., Murphy C., Fotsis T. (2013). Anti-angiogenesis in cancer therapy: hercules and hydra. *Cancer Letters*.

[5] Costa P. Z., Soares R. (2013). Neovascularization in diabetes and its complications. Unraveling the angiogenic paradox. *Life Sciences*.

[6] Zachary I., Morgan R. D. (2011). Therapeutic angiogenesis for cardiovascular disease: biological context, challenges, prospects. *Heart*.

[7] Asahara T., Murohara T., Sullivan A. (1997). Isolation of putative progenitor endothelial cells for angiogenesis. *Science*.

[8] Asahara T., Masuda H., Takahashi T. (1999). Bone marrow origin of endothelial progenitor cells responsible for postnatal vasculogenesis in physiological and pathological neovascularization. *Circulation Research*.

[9] Takahashi T., Kalka C., Masuda H. (1999). Ischemia- and cytokine-induced mobilization of bone marrow-derived endothelial progenitor cells for neovascularization. *Nature Medicine*.

[10] Richardson M. R., Yoder M. C. (2011). Endothelial progenitor cells: quo vadis?. *Journal of Molecular and Cellular Cardiology*.

[11] Kachamakova-Trojanowska N., Bukowska-Strakova K., Zukowska M., Dulak J., Jozkowicz A. (2015). The real face of endothelial progenitor cells—circulating angiogenic cells as endothelial prognostic marker?. *Pharmacological Reports*.

[12] Tamarat R., Silvestre J.-S., Le Ricousse-Roussanne S. (2004). Impairment in ischemia-induced neovascularization in diabetes: bone marrow mononuclear cell dysfunction and therapeutic potential of placenta growth factor treatment. *The American Journal of Pathology*.

[13] Zhang H.-F., Wang L., Yuan H.-J. (2013). PPAR-*γ* agonist pioglitazone prevents apoptosis of endothelial progenitor cells from rat bone marrow. *Cell Biology International*.

[14] Garcia-Barros M., Paris F., Cordon-Cardo C. (2003). Tumor response to radiotherapy regulated by endothelial cell apoptosis. *Science*.

[15] Salven P., Mustjoki S., Alitalo R., Alitalo K., Rafii S. (2003). VEGFR-3 and CD133 identify a population of CD34^+^ lymphatic/vascular endothelial precursor cells. *Blood*.

[16] Urbich C., Dimmeler S. (2004). Endothelial progenitor cells: functional characterization. *Trends in Cardiovascular Medicine*.

[17] Spring H., Schüler T., Arnold B., Hämmerling G. J., Ganss R. (2005). Chemokines direct endothelial progenitors into tumor neovessels. *Proceedings of the National Academy of Sciences of the United States of America*.

[18] Kopp H.-G., Ramos C. A., Rafii S. (2006). Contribution of endothelial progenitors and proangiogenic hematopoietic cells to vascularization of tumor and ischemic tissue. *Current Opinion in Hematology*.

[19] Purhonen S., Palm J., Rossi D. (2008). Bone marrow-derived circulating endothelial precursors do not contribute to vascular endothelium and are not needed for tumor growth. *Proceedings of the National Academy of Sciences of the United States of America*.

[20] Fadini G. P., Losordo D., Dimmeler S. (2012). Critical reevaluation of endothelial progenitor cell phenotypes for therapeutic and diagnostic use. *Circulation Research*.

[21] Yoder M. C. (2013). Endothelial progenitor cell: a blood cell by many other names may serve similar functions. *Journal of Molecular Medicine*.

[22] Urbich C., Aicher A., Heeschen C. (2005). Soluble factors released by endothelial progenitor cells promote migration of endothelial cells and cardiac resident progenitor cells. *Journal of Molecular and Cellular Cardiology*.

[23] Grochot-Przeczek A., Kotlinowski J., Kozakowska M. (2014). Heme oxygenase-1 is required for angiogenic function of bone marrow-derived progenitor cells: role in therapeutic revascularization. *Antioxidants and Redox Signaling*.

[24] Kotlinowski J., Grochot-Przeczek A., Taha H. (2014). PPAR*γ* activation but not PPAR*γ* haplodeficiency affects proangiogenic potential of endothelial cells and bone marrow-derived progenitors. *Cardiovascular Diabetology*.

[25] Chen F., Law S. W., O'Malley B. W. (1993). Identification of two mPPAR related receptors and evidence for the existence of five subfamily members. *Biochemical and Biophysical Research Communications*.

[26] Greene M. E., Blumberg B., McBride O. W. (1995). Isolation of the human peroxisome proliferator activated receptor gamma cDNA: expression in hematopoietic cells and chromosomal mapping. *Gene Expression*.

[27] Ahmadian M., Suh J. M., Hah N. (2013). PPAR*γ* signaling and metabolism: the good, the bad and the future. *Nature Medicine*.

[28] Sauer S. (2015). Ligands for the nuclear peroxisome proliferator-activated receptor gamma. *Trends in Pharmacological Sciences*.

[29] Chawla A., Schwarz E. J., Dimaculangan D. D., Lazar M. A. (1994). Peroxisome proliferator-activated receptor (PPAR) *γ*: adipose-predominant expression and induction early in adipocyte differentiation. *Endocrinology*.

[30] Fajas L., Auboeuf D., Raspé E. (1997). The organization, promoter analysis, and expression of the human PPAR*γ* gene. *Journal of Biological Chemistry*.

[31] Xin X., Yang S., Kowalski J., Gerritsen M. E. (1999). Peroxisome proliferator-activated receptor *γ* ligands are potent inhibitors of angiogenesis in vitro and in vivo. *The Journal of Biological Chemistry*.

[32] Marx N., Schönbeck U., Lazar M. A., Libby P., Plutzky J. (1998). Peroxisome proliferator-activated receptor gamma activators inhibit gene expression and migration in human vascular smooth muscle cells. *Circulation Research*.

[33] Barak Y., Nelson M. C., Ong E. S. (1999). PPAR*γ* is required for placental, cardiac, and adipose tissue development. *Molecular Cell*.

[34] Duan S. Z., Ivashchenko C. Y., Whitesall S. E. (2007). Hypotension, lipodystrophy, and insulin resistance in generalized PPAR*γ*-deficient mice rescued from embryonic lethality. *Journal of Clinical Investigation*.

[35] Nicol C. J., Adachi M., Akiyama T. E., Gonzalez F. J. (2005). PPAR*γ* in endothelial cells influences high fat diet-induced hypertension. *American Journal of Hypertension*.

[36] Kleinhenz J. M., Kleinhenz D. J., You S. (2009). Disruption of endothelial peroxisome proliferator-activated receptor-*γ* reduces vascular nitric oxide production. *American Journal of Physiology—Heart and Circulatory Physiology*.

[37] Nadra K., Quignodon L., Sardella C. (2010). PPAR*γ* in placental angiogenesis. *Endocrinology*.

[38] McCarthy F. P., Drewlo S., English F. A. (2011). Evidence implicating peroxisome proliferator-activated receptor-*γ* in the pathogenesis of preeclampsia. *Hypertension*.

[39] Bishop-Bailey D., Hla T. (1999). Endothelial cell apoptosis induced by the peroxisome proliferator-activated receptor (PPAR) ligand 15-deoxy-Δ^12,14^-prostaglandin J_2_. *The Journal of Biological Chemistry*.

[40] Ye P., Hu X., Zhao Y. (2002). The increase in plasminogen activator inhibitor type-1 expression by stimulation of activators for peroxisome proliferator-activated receptors in human endothelial cells. *Chinese Medical Sciences Journal*.

[41] Jözkowicz A., Huk I., Nigisch A. (2003). Heme oxygenase and angiogenic activity of endothelial cells: stimulation by carbon monoxide and inhibition by tin protoporphyrin-IX. *Antioxidants and Redox Signaling*.

[42] Kato K., Satoh H., Endo Y. (1999). Thiazolidinediones down-regulate plasminogen activator inhibitor type 1 expression in human vascular endothelial cells: a possible role for PPAR*γ* in endothelial function. *Biochemical and Biophysical Research Communications*.

[43] Liu H. B., Hu Y. S., Medcalf R. L., Simpson R. W., Dear A. E. (2005). Thiazolidinediones inhibit TNF*α* induction of PAI-1 independent of PPAR*γ* activation. *Biochemical and Biophysical Research Communications*.

[44] Józkowicz A., Huk I., Nigisch A., Cisowski J., Weigel G., Dulak J. (2003). Prostaglandin-J2 upregulates expression of matrix metalloproteinase-1 independently of activation of peroxisome proliferator-activated receptor-*γ*. *Acta Biochimica Polonica*.

[45] Park B. C., Thapa D., Lee J. S., Park S.-Y., Kim J.-A. (2009). Troglitazone inhibits vascular endothelial growth factor-induced angiogenic signaling via suppression of reactive oxygen species production and extracellular signal-regulated kinase phosphorylation in endothelial cells. *Journal of Pharmacological Sciences*.

[46] Huang W., Sung Y. E., András I. E., Hennig B., Toborek M. (2009). PPAR*α* and PPAR*γ* attenuate HIV-induced dysregulation of tight junction proteins by modulations of matrix metalloproteinase and proteasome activities. *The FASEB Journal*.

[47] Tyagi N., Moshal K. S., Sen U., Lominadze D., Ovechkin A. V., Tyagi S. C. (2006). Ciglitazone ameliorates homocysteine-mediated mitochondrial translocation and matrix metalloproteinase-9 activation in endothelial cells by inducing peroxisome proliferator activated receptor-*γ* activity. *Cellular and Molecular Biology*.

[48] Moens S., Goveia J., Stapor P. C., Cantelmo A. R., Carmeliet P. (2014). The multifaceted activity of VEGF in angiogenesis—implications for therapy responses. *Cytokine and Growth Factor Reviews*.

[49] Funovics P., Brostjan C., Nigisch A. (2006). Effects of 15d-PGJ_2_ on VEGF-induced angiogenic activities and expression of VEGF receptors in endothelial cells. *Prostaglandins & Other Lipid Mediators*.

[50] Kim K. Y., Ahn J. H., Cheon H. G. (2011). Anti-angiogenic action of PPAR*γ* ligand in human umbilical vein endothelial cells is mediated by PTEN upregulation and VEGFR-2 downregulation. *Molecular and Cellular Biochemistry*.

[51] Tian J., Smith A., Nechtman J. (2009). Effect of PPAR*γ* inhibition on pulmonary endothelial cell gene expression: gene profiling in pulmonary hypertension. *Physiological Genomics*.

[52] Fujii M., Inoki I., Saga M. (2012). Aldosterone inhibits endothelial morphogenesis and angiogenesis through the downregulation of vascular endothelial growth factor receptor-2 expression subsequent to peroxisome proliferator-activated receptor gamma. *Journal of Steroid Biochemistry and Molecular Biology*.

[53] Jozkowicz A., Dulak J., Piatkowska E., Placha W., Dembinska-Kiec A. (2000). Ligands of peroxisome proliferator-activated receptor-*γ* increase the generation of vascular endothelial growth factor in vascular smooth muscle cells and in macrophages. *Acta Biochimica Polonica*.

[54] Yamakawa K., Hosoi M., Koyama H. (2000). Peroxisome proliferator-activated receptor-*γ* agonists increase vascular endothelial growth factor expression in human vascular smooth muscle cells. *Biochemical and Biophysical Research Communications*.

[55] Jozkowicz A., Huk I., Nigisch A., Weigel G., Weidinger F., Dulak J. (2002). Effect of prostaglandin-J_2_ on VEGF synthesis depends on the induction of heme oxygenase-1. *Antioxidants & Redox Signaling*.

[56] Józkowicz A., Nigisch A., Wȩgrzyn J., Weigel G., Huk I., Dulak J. (2004). Opposite effects of prostaglandin-J2 on VEGF in normoxia and hypoxia: role of HIF-1. *Biochemical and Biophysical Research Communications*.

[57] Biscetti F., Gaetani E., Flex A. (2008). Selective activation of peroxisome proliferator-activated receptor (PPAR)*α* and PPAR*γ* induces neoangiogenesis through a vascular endothelial growth factor-dependent mechanism. *Diabetes*.

[58] Biscetti F., Straface G., Arena V. (2009). Pioglitazone enhances collateral blood flow in ischemic hindlimb of diabetic mice through an Akt-dependent VEGF-mediated mechanism, regardless of PPAR*γ* stimulation. *Cardiovascular Diabetology*.

[59] Fukunaga Y., Itoh H., Doi K. (2001). Thiazolidinediones, peroxisome proliferator-activated receptor *γ* agonists, regulate endothelial cell growth and secretion of vasoactive peptides. *Atherosclerosis*.

[60] Hong H. K., Cho Y. M., Park K.-H., Lee C.-T., Lee H. K., Park K. S. (2003). Peroxisome proliferator-activated receptor gamma mediated inhibition of plasminogen activator inhibitor type 1 production and proliferation of human umbilical vein endothelial cells. *Diabetes Research and Clinical Practice*.

[61] Desouza C. V., Gerety M., Hamel F. G. (2009). Effects of a PPAR-gamma agonist, on growth factor and insulin stimulated endothelial cells. *Vascular Pharmacology*.

[62] Sheu W. H.-H., Ou H.-C., Chou F.-P., Lin T.-M., Yang C.-H. (2006). Rosiglitazone inhibits endothelial proliferation and angiogenesis. *Life Sciences*.

[63] Lee K.-S., Park J.-H., Lee S., Lim H.-J., Jang Y., Park H.-Y. (2006). Troglitazone inhibits endothelial cell proliferation through suppression of casein kinase 2 activity. *Biochemical and Biophysical Research Communications*.

[64] Goetze S., Eilers F., Bungenstock A. (2002). PPAR activators inhibit endothelial cell migration by targeting Akt. *Biochemical and Biophysical Research Communications*.

[65] Goetze S., Bungenstock A., Czupalla C. (2002). Leptin induces endothelial cell migration through Akt, which is inhibited by PPAR*γ*-ligands. *Hypertension*.

[66] Huang J., Kontos C. D. (2002). PTEN modulates vascular endothelial growth factor-mediated signaling and angiogenic effects. *The Journal of Biological Chemistry*.

[67] Hannan K. M., Dilley R. J., de Dios S. T., Little P. J. (2003). Troglitazone stimulates repair of the endothelium and inhibits neointimal formation in denuded rat aorta. *Arteriosclerosis, Thrombosis, and Vascular Biology*.

[68] Aljada A., O'Connor L., Fu Y.-Y., Mousa S. A. (2008). PPAR*γ* ligands, rosiglitazone and pioglitazone, inhibit bFGF- and VEGF-mediated angiogenesis. *Angiogenesis*.

[69] Arnaoutova I., George J., Kleinman H. K., Benton G. (2009). The endothelial cell tube formation assay on basement membrane turns 20: state of the science and the art. *Angiogenesis*.

[70] Jiang X., Ye X., Guo W., Lu H., Gao Z. (2014). Inhibition of HDAC3 promotes ligand-independent PPAR*γ* activation by protein acetylation. *Journal of Molecular Endocrinology*.

[71] Murata T., He S., Hangai M. (2000). Peroxisome proliferator-activated receptor-*γ* ligands inhibit choroidal neovascularization. *Investigative Ophthalmology & Visual Science*.

[72] Jozkowicz A., Dulak J., Prager M. (2001). Prostaglandin-J_2_ induces synthesis of interleukin-8 by endothelial cells in a PPAR*γ*-independent manner. *Prostaglandins and Other Lipid Mediators*.

[73] Huang H., Campbell S. C., Bedford D. F. (2004). Peroxisome proliferator-activated receptor *γ* ligands improve the antitumor efficacy of thrombospondin peptide ABT510. *Molecular Cancer Research*.

[74] Chi J.-T., Chang H. Y., Haraldsen G. (2003). Endothelial cell diversity revealed by global expression profiling. *Proceedings of the National Academy of Sciences of the United States of America*.

[75] Nolan D., Ginsberg M., Israely E. (2013). Molecular signatures of tissue-specific microvascular endothelial cell heterogeneity in organ maintenance and regeneration. *Developmental Cell*.

[76] Trepels T., Zeiher A. M., Fichtlscherer S. (2006). The endothelium and inflammation. *Endothelium*.

[77] Zampetaki A., Kirton J. P., Xu Q. (2008). Vascular repair by endothelial progenitor cells. *Cardiovascular Research*.

[78] Florczyk U., Jazwa A., Maleszewska M. (2014). Nrf2 regulates angiogenesis: effect on endothelial cells, bone marrow-derived proangiogenic cells and hind limb ischemia. *Antioxidants & Redox Signaling*.

[79] Tepper O. M., Galiano R. D., Capla J. M. (2002). Human endothelial progenitor cells from type II diabetics exhibit impaired proliferation, adhesion, and incorporation into vascular structures. *Circulation*.

[80] Fadini G. P., Miorin M., Facco M. (2005). Circulating endothelial progenitor cells are reduced in peripheral vascular complications of type 2 diabetes mellitus. *Journal of the American College of Cardiology*.

[81] Fadini G. P., Sartore S., Schiavon M. (2006). Diabetes impairs progenitor cell mobilisation after hindlimb ischaemia-reperfusion injury in rats. *Diabetologia*.

[82] Nowak W. N., Borys S., Kusińska K. (2014). Number of circulating pro-angiogenic cells, growth factor and anti-oxidative gene profiles might be altered in type 2 diabetes with and without diabetic foot syndrome. *Journal of Diabetes Investigation*.

[83] Pistrosch F., Herbrig K., Oelschlaegel U. (2005). PPAR*γ*-agonist rosiglitazone increases number and migratory activity of cultured endothelial progenitor cells. *Atherosclerosis*.

[84] Wang C.-H., Ting M.-K., Verma S. (2006). Pioglitazone increases the numbers and improves the functional capacity of endothelial progenitor cells in patients with diabetes mellitus. *American Heart Journal*.

[85] Liang C., Ren Y., Tan H. (2009). Rosiglitazone via upregulation of Akt/eNOS pathways attenuates dysfunction of endothelial progenitor cells, induced by advanced glycation end products. *British Journal of Pharmacology*.

[86] Spigoni V., Picconi A., Cito M. (2012). Pioglitazone improves *in vitro* viability and function of endothelial progenitor cells from individuals with impaired glucose tolerance. *PLoS ONE*.

[87] Werner C., Kamani C. H., Gensch C., Böhm M., Laufs U. (2007). The peroxisome proliferator-activated receptor-*γ* agonist pioglitazone increases number and function of endothelial progenitor cells in patients with coronary artery disease and normal glucose tolerance. *Diabetes*.

[88] Gensch C., Clever Y. P., Werner C., Hanhoun M., Böhm M., Laufs U. (2007). The PPAR-*γ* agonist pioglitazone increases neoangiogenesis and prevents apoptosis of endothelial progenitor cells. *Atherosclerosis*.

[89] Werner C., Gensch C., Pöss J., Haendeler J., Böhm M., Laufs U. (2011). Pioglitazone activates aortic telomerase and prevents stress-induced endothelial apoptosis. *Atherosclerosis*.

[90] Sorrentino S. A., Bahlmann F. H., Besler C. (2007). Oxidant stress impairs in vivo reendothelialization capacity of endothelial progenitor cells from patients with type 2 diabetes mellitus: restoration by the peroxisome proliferator-activated receptor-*γ* agonist rosiglitazone. *Circulation*.

[91] Kirfel G., Rigort A., Borm B., Herzog V. (2004). Cell migration: mechanisms of rear detachment and the formation of migration tracks. *European Journal of Cell Biology*.

[92] Gealekman O., Guseva N., Gurav K. (2012). Effect of rosiglitazone on capillary density and angiogenesis in adipose tissue of normoglycaemic humans in a randomised controlled trial. *Diabetologia*.

[93] Werner C. M., Schirmer S. H., Gensch C. (2014). The dual PPAR*α*/*γ* agonist aleglitazar increases the number and function of endothelial progenitor cells: implications for vascular function and atherogenesis. *British Journal of Pharmacology*.

